# Combination CTLA-4 Blockade and 4-1BB Activation Enhances Tumor Rejection by Increasing T-Cell Infiltration, Proliferation, and Cytokine Production

**DOI:** 10.1371/journal.pone.0019499

**Published:** 2011-04-29

**Authors:** Michael A. Curran, Myoungjoo Kim, Welby Montalvo, Aymen Al-Shamkhani, James P. Allison

**Affiliations:** 1 Howard Hughes Medical Institute, Department of Immunology, Memorial Sloan-Kettering Cancer Center, New York, New York, United States of America; 2 Cancer Sciences Division, University of Southampton School of Medicine, Southampton General Hospital, Southampton, United Kingdom; La Jolla Institute of Allergy and Immunology, United States of America

## Abstract

**Background:**

The co-inhibitory receptor Cytotoxic T-Lymphocyte Antigen 4 (CTLA-4) attenuates immune responses and prevent autoimmunity, however, tumors exploit this pathway to evade the host T-cell response. The T-cell co-stimulatory receptor 4-1BB is transiently upregulated on T-cells following activation and increases their proliferation and inflammatory cytokine production when engaged. Antibodies which block CTLA-4 or which activate 4-1BB can promote the rejection of some murine tumors, but fail to cure poorly immunogenic tumors like B16 melanoma as single agents.

**Methodology/Principal Findings:**

We find that combining αCTLA-4 and α4-1BB antibodies in the context of a Flt3-ligand, but not a GM-CSF, based B16 melanoma vaccine promoted synergistic levels of tumor rejection. 4-1BB activation elicited strong infiltration of CD8+ T-cells into the tumor and drove the proliferation of these cells, while CTLA-4 blockade did the same for CD4+ effector T-cells. Anti-4-1BB also depressed regulatory T-cell infiltration of tumors. 4-1BB activation strongly stimulated inflammatory cytokine production in the vaccine and tumor draining lymph nodes and in the tumor itself. The addition of CTLA-4 blockade further increased IFN-γ production from CD4+ effector T-cells in the vaccine draining node and the tumor. Anti 4-1BB treatment, with or without CTLA-4 blockade, induced approximately 75% of CD8+ and 45% of CD4+ effector T-cells in the tumor to express the killer cell lectin-like receptor G1 (KLRG1). Tumors treated with combination antibody therapy showed 1.7-fold greater infiltration by these KLRG1+CD4+ effector T-cells than did those treated with α4-1BB alone.

**Conclusions/Significance:**

This study shows that combining T-cell co-inhibitory blockade with αCTLA-4 and active co-stimulation with α4-1BB promotes rejection of B16 melanoma in the context of a suitable vaccine. In addition, we identify KLRG1 as a useful marker for monitoring the anti-tumor immune response elicited by this therapy. These findings should aid in the design of future trials for the immunotherapy of melanoma.

## Introduction

The co-inhibitory receptor Cytotoxic T-Lymphocyte Antigen 4 (CTLA-4) is induced on T-cells shortly after activation and functions to attenuate their proliferation, IL-2 production, and contact time with antigen presenting cells (APC) [1], [2]. Also, CTLA-4 appears to support the function of the regulatory T-cell (Treg) compartment [3]. Antibody blockade of CTLA-4 removes these suppressive signals and allows tumor-specific T-cells which would otherwise be anergized to expand and continue to perform effector functions. Previously, we have shown that the therapeutic efficacy of CTLA-4 blockade against poorly immunogenic tumors like B16 melanoma is strongly enhanced by co-administration of an autologous tumor vaccine expressing either the cytokine Granulocyte-macrophage colony-stimulating factor (GM-CSF) or FMS-like tyrosine kinase 3 ligand (Flt3-ligand) [4], [5].

4-1BB (CD137) belongs to the Tumor Necrosis Factor Receptor (TNFR) superfamily and is transiently upregulated on both CD4+ and CD8+ T-cells following activation [6]. 4-1BB ligation is known to co-stimulate CD8+ T-cells increasing their proliferation, TH1 cytokine production, and survival [7]. A majority of Tregs express 4-1BB, but it remains unclear whether agonist antibody treatment exerts a pro- or anti-suppressive effect on these cells [8], [9], [10], [11]. In immunotherapy studies, 4-1BB antibodies can enhance tumor rejection, increase tumor-specific cytotoxicity, and may render effector T-cells resistant to Treg suppression [10], [12], [13], [14], [15]. The mechanisms underlying many of these observed anti-tumor effects, however, remain to be elucidated.

Prior studies have shown that agonistic 4-1BB antibodies with or without CTLA-4 blockade can promote the rejection of some murine tumors and ameliorate auto-immune toxicity; however, poorly immunogenic tumors such as B16 melanoma do not respond to antibody therapy alone [10], [14]. We hypothesized that increasing the tumor-specific T-cell frequency through vaccination would allow us to better observe the interaction between 4-1BB activation and CTLA-4 blockade in the B16 melanoma system.

We found that α4-1BB and αCTLA-4 synergized in rejecting pre-implanted B16 melanomas in conjunction with a B16-Flt3-ligand (FVAX) but not a B16-GMCSF (GVAX) vaccine. Combination therapy yielded highly advantageous ratios of intra-tumoral CD8+ and CD4+ effector T-cells relative to Tregs which often correlates with rejection in this system [4], [16]. 4-1BB agonist antibody promoted very high levels of both peripheral and intra-tumoral inflammatory cytokine production; however, the addition of CTLA-4 blockade strongly augmented IFN-γ production from CD4+ effector cells. Likewise, αCTLA-4 played a critical role in allowing proliferation of tumor infiltrating CD4+ effectors.

4-1BB agonist antibody treatment induced widespread expression of the molecule killer cell lectin-like receptor subfamily G member 1 (KLRG1) as well as the co-inhibitory receptor programmed death 1 (PD-1) on tumor-infiltrating CD8+ and CD4+ effector T-cells. We observed the highest numbers of these KLRG1+ effector T-cells in the tumors of mice receiving combination CTLA-4 blockade and 4-1BB agonist antibody suggesting a positive correlation with therapeutic outcome.

By combining T-cell co-inhibitory blockade with co-stimulatory activation, we were able to induce rejection of poorly immunogenic B16 melanoma tumors. Also, we have characterized the cellular and molecular mechanisms driving this response further clarifying the basic processes necessary to achieve immune-mediated tumor rejection.

## Results

### 4-1BB agonist antibody treatment cooperates with CTLA-4 blockade and B16-Flt3-ligand vaccination to promote B16 melanoma rejection

**Figure 1:**
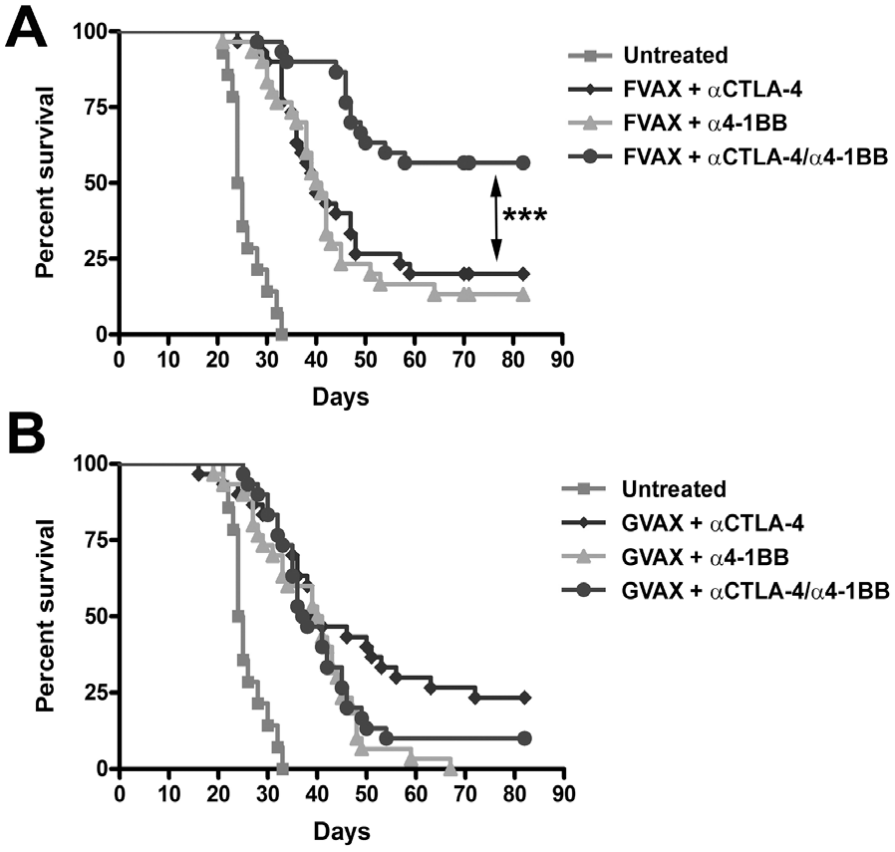
Anti-CTLA-4 and α4-1BB synergize in the context of FVAX. Kaplan-Meier survival curves for mice challenged with 2.5×10^4^ B16-BL6 cells and vaccinated on days 3, 6 and 9 with 1×10^6^ of A) FVAX or B) GVAX intra-dermally and the indicated antibody or combination intra-peritoneally. Lack of survival was defined as death or tumor size >1000 mm^3^. Each curve represents 3 independent experiments of 10 mice per group. P values were calculated using the Log-rank (Mantel-Cox) test (* - p≤0.05, ** - p≤0.01, ***-p<0.001).

Mice were challenged with B16-BL6 melanoma and vaccinated on days 3, 6, and 9 with α4-1BB and/or αCTLA-4 in addition to either GVAX, FVAX, or no vaccine. As others have seen in the B16-F10 model, we observed no curative effect of the antibodies without cellular vaccination (Figure S1) [10], [14]. When administered with FVAX, combination co-stimulatory modulation promoted rejection in 57% of B16 melanoma challenged mice versus 20% and 13% for αCTLA-4 and α4-1BB alone (Figure 1A). In the case of GVAX, however, combination therapy with αCTLA-4 and α4-1BB was no better than αCTLA-4 alone (Figure 1B).

### Combination αCTLA-4 and α4-1BB therapy strongly increases both CD8+/Treg and CD4+ effector T-cell/Treg ratios within the tumor

**Figure 2:**
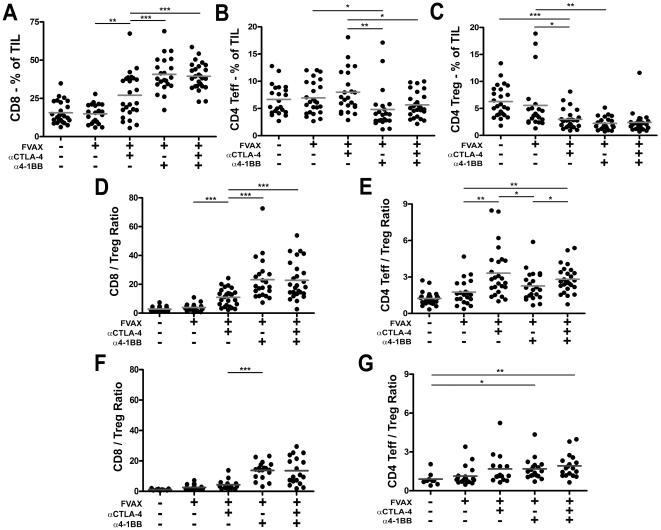
Combination αCTLA-4/α4-1BB therapy promotes high effector to regulatory T-cell ratios in the tumor. Mice were challenged with 1.5×10^5^ B16-BL6, treated with FVAX and the indicated antibody on days 3,6 and 9, and sacrificed on day 15. Percent of CD45+ tumor infiltrating lymphocytes of A) CD8+ T-cells, B) CD4+ effector (Teff) and C) regulatory (Treg) cells is shown. The ratios of D) CD8+ T-cells to Tregs and E) CD4+ Teff to Treg are shown. Values shown are for individually analyzed mice and are the sum of 5 independent experiments with 5–15 mice per group. The ratios of F) CD8+ T-cells to Tregs and G) CD4+ Teff to Treg are shown at Day 25 for individual mice from 2 independent experiments. Student's t-tests were performed to determine statistical significance between samples (* - p≤0.05, ** - p≤0.01, ***-p<0.001).

To understand the apparent synergy between CTLA-4 blockade and 4-1BB activation in the context of our Flt3-ligand base vaccine, we sought to dissect the effects of each therapy on T-cell infiltration of tumor in this background. 4-1BB activation promoted very strong CD8 infiltration of B16 melanoma, but in doing so depressed the relative fraction of CD4+ effector cells (Figure 2A and B). In terms of absolute T-cell numbers, the CD8 T-cell density increased while the CD4 effector T-cell levels were unchanged (Figure S2A and B). In contrast to α4-1BB, blockade of CTLA-4 increased both the TIL fraction and absolute numbers of CD4+ effector T-cells infiltrating the tumors. Combination therapy with both antibodies benefits from the highly elevated CD8+ infiltration evoked by α4-1BB, complemented by the CD4+ effector T-cell infiltration elicited by αCTLA-4.

While both co-stimulatory modulating antibodies depressed the fraction of Tregs in B16 melanoma TIL (Figure 2C), CTLA-4 blockade increased absolute numbers of Tregs in the tumor at day 14, whereas α4-1BB did not (Figure S2C). Together, αCTLA-4 and α4-1BB not only diminish the Treg fraction of TIL, but also reduce the intra-tumoral numbers of Tregs to those achieved with α4-1BB alone.

By increasing CD8 infiltration and attenuating regulatory T-cell accumulation, combination αCTLA-4 and α4-1BB treatment strongly increased intra-tumoral CD8 to Treg ratios (Figure 2D). CD4 effector to Treg ratios in the tumor were also elevated in combination therapy, largely due to the effects of CTLA-4 blockade in promoting increased CD4 infiltration (Figure 2E). We conclude that the combination therapy creates higher CD8/Treg ratios than CTLA-4 blockade alone (23∶1 versus 11∶1, p = 0.0002), while also providing a significantly higher CD4 T-effector/Treg ratio compared to either α4-1BB alone (2.8∶1 versus 2∶1, p = 0.027) or to FVAX alone (2.8∶1 versus 1.8∶1, p = 0.0077) which α4-1BB therapy alone lacks. This combination of both high CD8 and CD4 effector to Treg ratios within the tumor represents the conversion of the normally suppressive tumor microenvironment to a more pro-inflammatory state which is more permissive for immune-mediated tumor rejection.

The rapidly progressive nature of B16 melanoma makes it difficult to assay tumor infiltrates at later time points than Day-15, however by using an asymmetric tumor challenge we were able to ask whether these enhanced effector to regulatory cell ratios were maintained. Even when the combination treated mice are given a 10-fold greater tumor challenge than the mice receiving FVAX alone, high CD8 to Treg ratios are evident at Day-25 in the groups receiving α4-1BB (Figure 2F). In contrast to Day-15, the CD4 effector to Treg ratios in the groups receiving α4-1BB equal those of αCTLA-4 treated mice by Day-25 (Figure 2G). This could represent the long term effects of the previously published pro-survival effects of 4-1BB agonist antibody. In all cases the effector to Treg ratios at Day-25 are diminished relative to their Day-15 counterparts. This is likely due to the increased time since the last vaccination, as well as the reality that all mice with sizable tumors at Day-25 have failed to respond at a therapeutic level to the treatment.

The failure of GVAX to support cooperativity between CTLA-4 blockade and 4-1BB activation does not appear to result from an inability to generate advantageous effector to Treg ratios. Although the CD8/Treg ratios generated in the context of GVAX are slightly lower than those with FVAX (Figure S3A), these differences do not appear substantial enough to explain the lack therapeutic synergy. Also, the CD4 effector to Treg ratios generated following treatment with α4-1BB and either GVAX or FVAX appear similar (Figure S3B).

### Combination αCTLA-4 and α4-1BB therapy increases inflammatory cytokine production in the vaccine and tumor draining lymph nodes, as well as in the tumor itself

**Figure 3:**
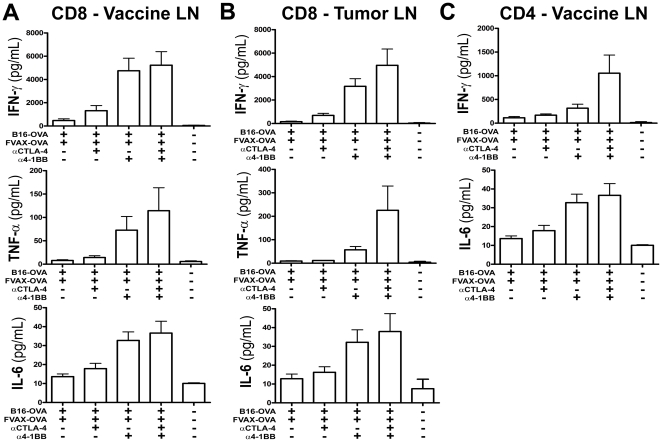
4-1BB activation cooperates with CTLA-4 blockade to induce peripheral inflammatory cytokine production. Mice challenged with 2.5×10^5^ B16-OVA cells and treated on days 6, 9 and 12, were sacrificed on Day 14. T-cells were purified from tumor-draining (TDLN) and vaccine-draining (VDLN) lymph nodes, stained with antibodies, and sorted by flow cytometry into CD4+ and CD8+ subsets. Cytokine production was measured after 36 hours using the TH1/TH2/TH17 CBA Kit (BD) and is shown for A) 2×10^5^ VDLN and B) 2×10^5^ TDLN CD8 T-cells restimulated on 1×10^5^ OVA 257–264 peptide pulsed DCs and for C) 2.5×10^5^ CD4 T-cells restimulated on 1×10^5^ OVA 323–339 peptide pulsed DCs. Data is shown for 3–4 independent experiments with 5–10 pooled mice per group.

To clearly define the functional effects of each therapy on effector cytokine production, we elected to use the B16-Ovalbumin (B16-Ova) model antigen system to measure cytokine responses. Mice were challenged with 2.5×10^5^ B16-Ova cells and vaccinated on days 6, 9, and 12 - cytokine production was analyzed on day 14. While CTLA-4 blockade did increase IFN-γ production from CD8 T-cells in the vaccine and tumor draining lymph nodes, α4-1BB evoked much stronger increases in IFN-γ, TNF-α, and IL-6 production (Figure 3A and B). In both nodes it appeared that αCTLA-4 and α4-1BB co-operated in driving higher levels of TNF-α production from CD8 T-cells. While we did not observe changes in TNF-α production from peripheral CD4+ effector T-cells, there appeared to be a synergistic effect of these two co-stimulatory modulating agents in promoting high IFN-γ production from CD4 cells in the vaccine draining node (Figure 3C). In contrast, we observed only low levels of IFN-γ production from CD4+ T-cells in the tumor draining lymph node (Figure S4). This >10-fold drop in IFN-γ production in the tumor-draining relative to the vaccine-draining lymph node suggested that the most active, tumor-specific CD4+ T-cells had already trafficked into the tumor. Therefore we asked whether inflammatory cytokine production from the tumor infiltrating T-cells had been altered by these therapies.

**Figure 4:**
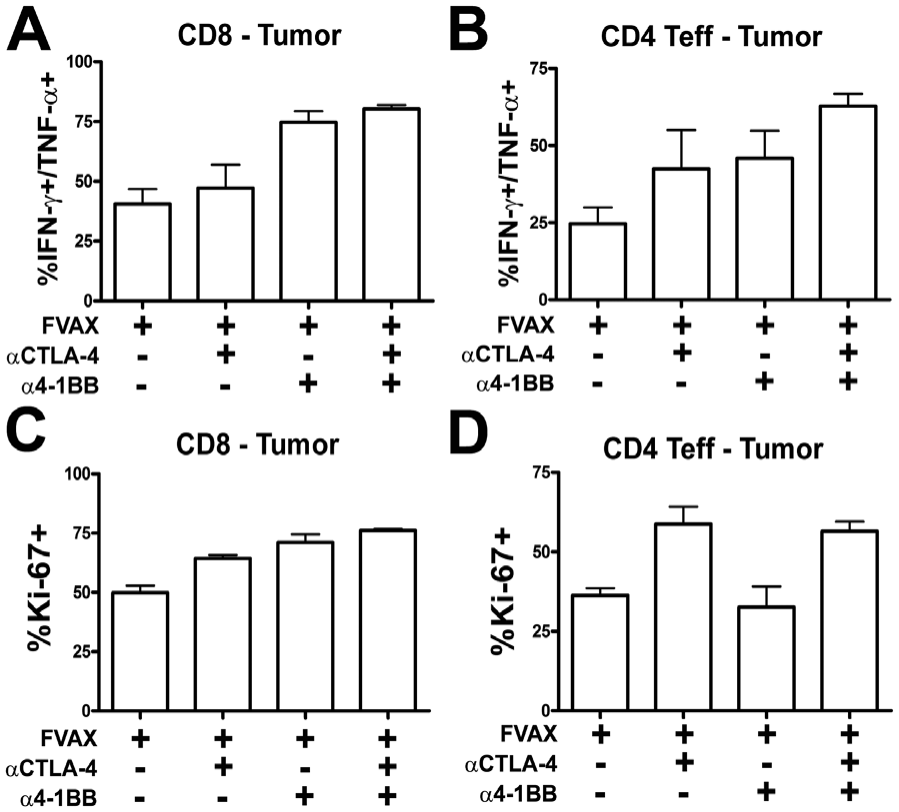
Combination αCTLA-4/α4-1BB therapy enhances intra-tumoral T-cell cytokine production and proliferation. Mice challenged with 2.5×10^5^ B16-OVA cells and treated on days 6, 9 and 12, were sacrificed on Day 14. TIL were purified from 5–10 pooled tumors per group and enriched using the Miltenyi T-cell purification kit. 2×10^6^ TIL were restimulated with 7.5×10^5^ DC (1∶1 mix of MHC-I and MHC-II peptide pulsed DCs) for 8 hours in the presence of BD GolgiPlug. Cells were fixed using the FoxP3 kit and analyzed by flow cytometry for lymphocyte markers and intracellular IFN-γ and TNF-α production for A) CD8s and B) CD4 Teffs and also Ki67 expression for C) CD8s and D) CD4 Teffs. Data shown is from 4 independent experiments. All means shown are +/− S.E.M.

We re-stimulated tumor infiltrating T-cells for 8 hours with both Ovalbumin and B16 peptide pulsed DCs and measured intra-cellular cytokine accumulation by flow cytometry (Figure S5). As in the periphery, α4-1BB promoted the greatest increase in IFN-γ, TNF-α double producing CD8+ T-cells in the tumor (Figure 4A). It also appeared that CTLA-4 blockade provided a small additive increase in this population. Both 4-1BB activation and αCTLA-4 augmented the inflammatory cytokine production from CD4+ effector T-cells, and their effects were additive when given in combination (Figure 4B). The combination of co-stimulatory modulation and FVAX yielded high levels of inflammatory cytokine production both in the periphery and in the tumor. We next asked whether these agents had an equally complementary effect on the proliferation of tumor-infiltrating T-cells.

Both CTLA-4 blockade and 4-1BB stimulation resulted in higher CD8+ T-cell proliferation within the tumor as measured by expression of the cell cycle associated protein Ki-67 (Figure 4C). In contrast to the CD8 compartment, only CTLA-4 blockade appeared capable of driving CD4+ effector T-cell proliferation within the tumor (Figure 4D). Interestingly, α4-1BB appeared to decrease the proliferation of tumor-infiltrating Tregs, but much of this effect was lost in combination with CTLA-4 blockade (Figure S6).

### Combination 4-1BB activation and CTLA-4 blockade induces high expression of KLRG1 and PD-1 on tumor-infiltrating effector T-cells

**Figure 5:**
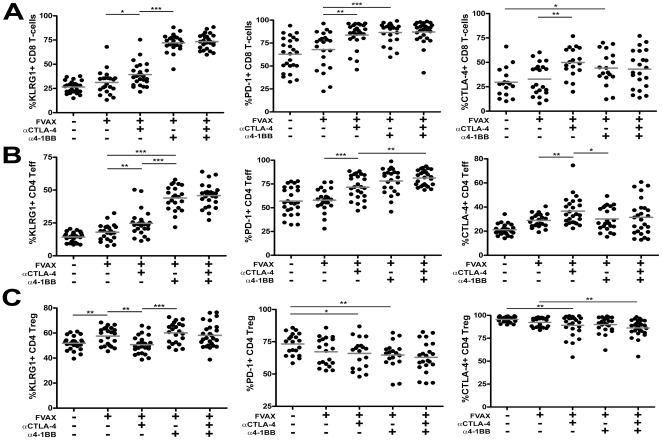
Combination αCTLA-4/α4-1BB therapy induces KLRG1 and PD-1 expression by tumor-infiltrating T-cells. Mice challenged with 1.5×10^5^ B16-BL6 cells and treated on days 3, 6 and 9, were sacrificed on Day 15. TIL were fixed and stained for lymphocyte lineage and activation markers using the FoxP3 fixation kit. Percent of A) CD8+ T-cells, B) CD4+ effector (Teff) and C) regulatory (Treg) cells expressing KLRG1, PD-1, and CTLA-4 are shown. The 4F10 clone of αCTLA-4 was used to detect cells on which CTLA-4 had been blocked *in vivo* using the 9D9 clone. Values shown are for individually analyzed mice and are the sum of 4–6 independent experiments with 5–15 mice per group. Student's t-tests were performed to determine statistical significance between samples(* - p≤0.05, ** - p≤0.01, ***-p<0.001).

We examined co-inhibitory and activation marker expression on tumor infiltrating T-cells following each therapy in an effort to gain further mechanistic insight into how these agents promoted tumor rejection. On tumor infiltrating CD8+ T-cells, there was a striking upregulation of PD-1 and of the surface receptor KLRG1 in response to both α4-1BB and combination antibody treatment(Figure 5A). KLRG1 expressing CD8+ T-cells have been previously described as a terminal effector population sometimes retained during clearance of viral infections [17]. CTLA-4 expression was increased on CD8+ T-cells in response to α4-1BB treatment, indicating these cells may have been saved from co-inhibitory attenuation in the combination therapy group.

Like the CD8 T-cells, the effector CD4+ T-cells infiltrating B16 melanoma expressed much higher levels of KLRG1 and PD-1 in response to α4-1BB or combination therapy (Figure 5B). In the CD4+ effector T-cells, however, CTLA-4 expression did not increase in response to α4-1BB.

Both 4-1BB activation and CTLA-4 blockade decreased CTLA-4 and PD-1 expression by Tregs in the tumor (Figure 5C). Unlike the tumor infiltrating effector T-cells, KLRG1 expression on Tregs was not significantly elevated in combination treated mice relative to those receiving FVAX alone.

Given the unique nature of this KLRG1 upregulation, we assayed whether mice treated with these antibodies in the context of GVAX rather than FVAX would generate a similar phenotype. We did observe an enhanced percentage of these KLRG1+ effector T-cells in the tumors of α4-1BB and αCTLA-4/α4-1BB treated mice receiving GVAX; however, the fraction of KLRG1+ T-cells in both compartments was lower than that generated with FVAX(Figure S7A and S7B).

### The KLRG1+ fraction of tumor infiltrating T-cells increases in 4-1BB agonist antibody treated mice by Day-25 relative to Day-15

**Figure 6:**
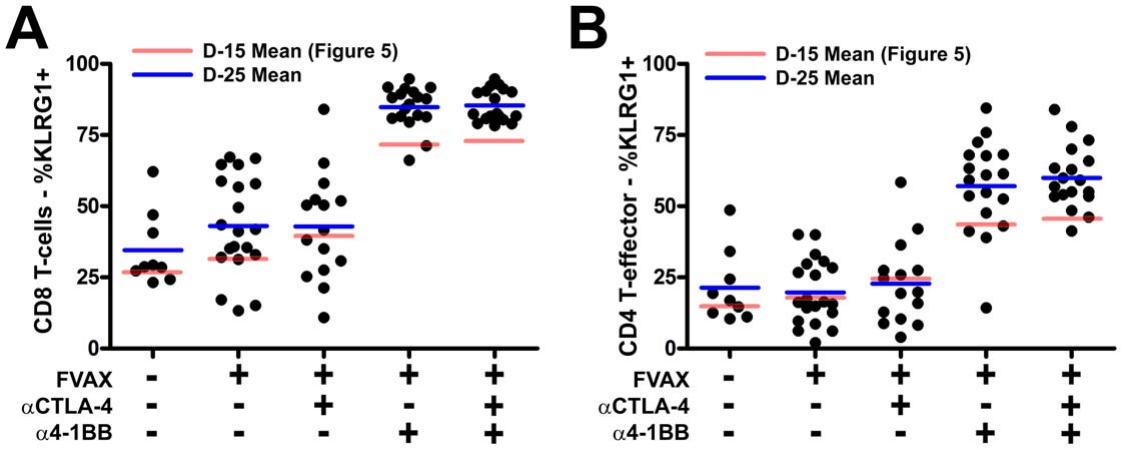
High KLRG1 expression by tumor-infiltrating effector T-cells at Day-25 in mice treated with combination αCTLA-4/α4-1BB therapy. Mice challenged with either 2.5×10^4^ (Untreated and FVAX), 1.0×10^5^ (FVAX+αCTLA-4 or α4-1BB), or 2.0×10^5^ (FVAX+αCTLA-4/α4-1BB) B16-BL6 cells and treated on days 4, 7 and 10, and 13 were sacrificed on Day 25. TIL were fixed and stained for lymphocyte lineage and activation markers using the FoxP3 fixation kit. Percent of A) CD8+ T-cells, B) CD4+ effector (Teff) expressing KLRG1 are shown. Values shown are for individually analyzed mice and are the sum of 2 independent experiments with 10 mice per group. Student's t-tests were performed to determine statistical significance between samples(* - p≤0.05, ** - p≤0.01, ***-p<0.001).

By Day-25 nearly all the CD8+ T-cells and the majority of CD4+ effector T-cells infiltrating the B16 melanoma tumors of α4-1BB and α4-1BB/αCTLA-4 treated mice are KLRG1+ (Figure 6A and B). This suggests either selective expansion and/or recruitment of these cells within the tumor microenvironment, or establishment of a cytokine environment capable of converting the majority of tumor infiltrating T-cells to the KLRG1+ phenotype.

### Tumors of mice receiving combination CTLA-4 blockade and 4-1BB activation are the most highly infiltrated by KLRG1+CD4+ effector T-cells

**Figure 7:**
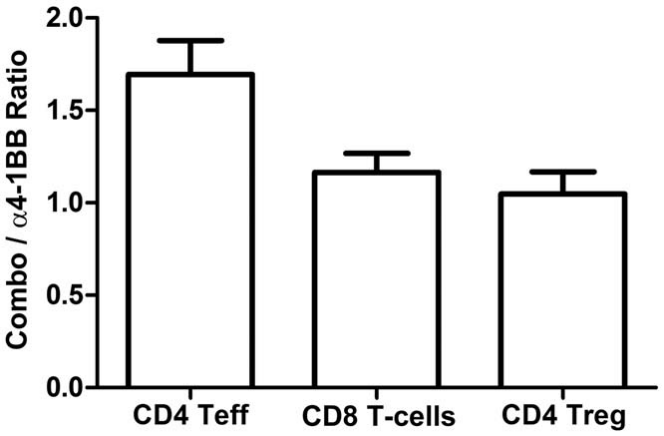
Combination αCTLA-4/α4-1BB therapy induces higher tumor infiltration by CD4+KLRG1+ T-cells than α4-1BB alone. Mice challenged with 1.5×10^5^ B16-BL6 cells and treated on days 3, 6 and 9, were sacrificed on Day 15. The number of KLRG1+ T-cells of a given lineage was calculated by multiplying the %KLRG1+ TIL determined by flow cytometry by the measured number of CD45+ lymphocytes per mm^3^ of tumor. Data shown are ratios of the absolute number of KLRG1+ T-cells from combination treated tumors to the number of KLRG1+ T-cells from α4-1BB alone treated tumors. Ratios were calculated for 5 independent experiments with 5–15 mice per group.

The combination of αCTLA-4 and α4-1BB demonstrated significantly higher therapeutic efficacy than α4-1BB alone (Figure 1); however, both groups show equally high increases in the frequency of tumor-infiltrating KLRG1+ T-cells in response to therapy (Figure 5A&B). By examining the absolute number of infiltrating KLRG1+ T-cells, however, we found that combination therapy results in 1.7-fold more CD4+KLRG1+ effector T-cells per mm^3^ of tumor relative to α4-1BB alone (Figure 7). By contrast, the number of KLRG1+ Tregs between these two groups was equal.

While in the FVAX background, more KLRG1+ effector T-cells correlated with superior therapeutic outcome, in the GVAX setting this was not the case. Although the tumors of mice treated with GVAX + αCTLA/α4-1BB versus those receiving GVAX + α4-1BB alone were infiltrated by over 6-fold more KLRG1+ CD8+ and CD4+ effector T-cells, these provided no additional therapeutic benefit (Figure S8). In contrast to the FVAX setting, however, the numbers of KLRG1+ Treg cells also increase over 4-fold in the context of GVAX. This complicates any interpretation of the potential therapeutic benefit of these KLRG1+ cells in the GVAX setting, as the KLRG1+ Treg subset has been previously described as a highly suppressive one [18].

### FVAX promotes more pro-inflammatory ratios of effector T-cells relative to CD11b+GR-1+ myeloid derived suppressor cells (MDSC) in antibody-treated mice than does GVAX

**Figure 8:**
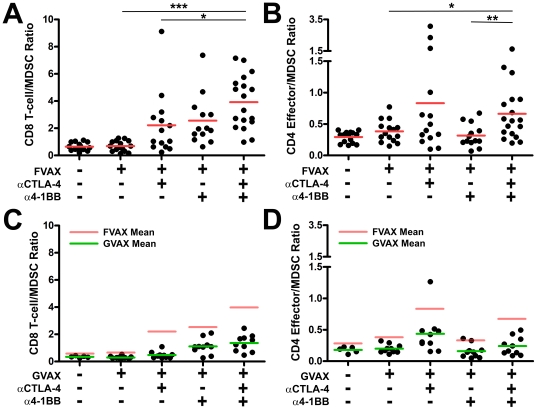
Combination αCTLA-4/α4-1BB treatment generates more pro-inflammatory ratios of effector T-cells to MDSC in the context of FVAX versus GVAX. Mice challenged with 1.5×10^5^ B16-BL6 cells and treated on days 3, 6 and 9, were sacrificed on Day 15. TIL were fixed and stained for myeloid and lymphocyte lineage and activation markers using the FoxP3 fixation kit. The ratios of A) CD8+ T-cells to CD11b+GR-1+ MDSC and B) CD4+ Teff to MDSC are shown in the FVAX setting. Values shown are for individually analyzed mice and are the sum of 3 independent experiments with 5–15 mice per group. The ratios of C) CD8+ T-cells to MDSC and D) CD4+ effector T-cells to MDSC are shown in the context of GVAX for 10 individual mice per group. Student's t-tests were performed to determine statistical significance between samples (* - p≤0.05, ** - p≤0.01, ***-p<0.001).

Mice receiving FVAX and both αCTLA-4 and α4-1BB show enhanced ratios of CD8+ T-cells relative to CD11b+GR-1+ MDSC when compared to either FVAX (3.9 vs. 0.7, p<0.0001) or αCTLA-4 alone (3.9 vs. 2.2, p = 0.03) (Figure 8A). Combination treated mice also exhibit enhanced ratios of CD4+FoxP3- effector T-cells relative to MDSC when compared to either FVAX (0.66 vs. 0.38, p = 0.02) or a4-1BB alone (0.66 vs. 0.31, p = 0.01) (Figure 8B). Thus, combination treatment with CTLA-4 blockade and 4-1BB agonist antibody promotes more pro-inflammatory ratios of CD8+ T-cells to MDSC than αCTLA-4 alone and higher CD4+ effector T-cell to MDSC ratios than α4-1BB alone.

GVAX has a known capacity to support the generation of MDSC even when injected on the opposite flank from tumor implantation [4], [19]. We hypothesized that a higher MDSC density in mice receiving GVAX versus FVAX might be responsible for the lack of synergy between CTLA-4 blockade and 4-1BB activation in the GVAX setting. In combination treated mice we observe both less advantageous CD8+ T-cell to MDSC ratios (1.4 vs. 6.9) and CD4+FoxP3- effector T-cell to MDSC ratios (0.24 vs. 0.66) with GVAX versus FVAX (Figure 8C&D). With GVAX there is also no significant difference between combination therapy and α4-1BB alone in terms of CD4 effector to MDSC ratio which may contribute to the lack of therapeutic cooperativity in this setting.

## Discussion

Antibodies which activate the T-cell co-stimulatory receptor 4-1BB or block the co-inhibitory receptor CTLA-4, have demonstrated broad anti-tumor effects but cannot induce rejection of the poorly immunogenic B16-BL6 melanoma without the aid of additional therapeutic interventions [10], [14]. In contrast to the more immunogenic B16-F10 model [14], we show that α4-1BB mediates few rejection of the highly aggressive B16-BL6 melanoma even in conjunction with GVAX(Figure 1). As we have observed previously for combination blockade of CTLA-4 and PD-1, we find that co-administration with an autologous tumor vaccine expressing Flt3-ligand allows these antibodies to co-operate in rejecting more than 50% of pre-implanted B16-BL6 melanomas [20]. Using this system, we sought to understand the impact on tumor-specific T-cell responses of simultaneously removing a major brake on expansion via blockade of the co-inhibitory receptor CTLA-4, while at the same time actively driving proliferation and survival through activation of the co-stimulatory receptor 4-1BB.

4-1BB expression is more pronounced on CD8 versus CD4 T-cells and for this reason most tumor immunity studies have focused on the impact of α4-1BB on this subset [6]. We found that 4-1BB activation promoted much stronger CD8 infiltration of tumors (Figure 2), inflammatory cytokine production by peripheral CD8 cells (Figure 3), and proliferation of tumor infiltrating CD8s (Figure 4). While CD4 effector infiltration and proliferation were only increased by CTLA-4 blockade (Figure 4), α4-1BB did increase inflammatory cytokine production from these cells both in the periphery and within the tumor itself. The mechanisms underlying this uncoupling of the proliferative and TH1 cytokine promoting effects of 4-1BB agonist antibody treatment in CD4 versus CD8 cells are of interest to us and will be addressed in future studies.

The activation of CD8 T-cells by α4-1BB coupled with the expansion of CD4 effector T-cells by αCTLA-4 clearly accounts for some of the observed synergy between these agents in rejecting B16 melanomas. 4-1BB activation has been described as a powerful promoter of TH1-type cytokine production [6], [21], [22]. In prior studies using the B16-Ova model, we have observed a tendency of co-inhibitory blockade to increase TH2 responses in the vaccine draining lymph node [20]. In this case, however, α4-1BB polarized cytokine production to TH1 and induced TNF-α production by CD8 T-cells and IFN-γ production by CD4 cells to levels that exceeded the sum of each individual therapy (Figure 3). Similarly, combination therapy additively increased the percentage of IFN-γ,TNF-α double producing CD4+ effectors in the tumor (Figure 4). A portion of the anti-tumor effect of CTLA-4 blockade derives from inhibition of Tregs [23]; however, the effects of α4-1BB on Treg proliferation and suppression remain unclear [8], [9], [10], [11]. Here, α4-1BB dampened Treg proliferation (Figure S6), reduced the Treg fraction of TIL (Figure 2), counteracted αCTLA-4's expansion of absolute Treg numbers in the tumor (Figure S2), and decreased CTLA-4 and PD-1 expression by Tregs (Figure 5). We conclude that at least a portion of the benefit of combination co-stimulatory modulation is due to reduction of Treg suppression in the tumor.

Distinct from other co-stimulatory antibodies, we found that α4-1BB induced striking upregulation of KLRG1 on CD8+, and to a lesser degree CD4+, effector T-cells in the tumor (Figure 5 and 6). This KLRG1 upregulation appeared unique to 4-1BB agonist antibody, as we did not observe a similar phenotype in response to αCTLA-4 (Figure 5), αPD-1, or αPD-L1 (Figure S9). We found that mice receiving the therapeutically more effective αCTLA-4/α4-1BB combination therapy had 1.7-fold more CD4+KLRG1+ cells infiltrating their tumors relative to mice treated with α4-1BB alone (Figure 7), suggesting a possible functional significance to this population. In addition, the KLRG1+ fraction of TIL seemed to increase over time for both CD8+ and CD4+ effector T-cells suggesting either enhanced infiltration, survival, or proliferation of these cells (Figure 6). The phenotype of these KLRG1+ tumor-infiltrating T-cells and their contribution to tumor rejection is of intense interest to us and will be the focus of future study.

The co-inhibitory receptor PD-1 which we have previously shown to be induced on T-cells following CTLA-4 blockade [20], also appears highly induced by 4-1BB activation (Figure 5A and B). Like CTLA-4, PD-1 can attenuate effector T-cell proliferation and effector function, causing anti-tumor immune responses to fail. The data presented here suggests that combining antibody blockade of PD-1 or both CTLA-4 and PD-1 with activation of 4-1BB may provide substantial benefit to auto-tumor immune responses.

Although our focus was on understanding the mechanisms underlying the therapeutic efficacy of combination αCTLA-4/α4-1BB treatment in the context of FVAX, we did explore potential reasons for the failure of GVAX to support synergy between these antibodies (Figure 1). While ratios of CD8 T-cells to Tregs within tumors of treated mice were slightly lower in the context of GVAX versus FVAX, the overall pattern of CD8 and CD4 effector T-cell to Treg ratios was similar in both treatment settings (Figure S3). Combination therapy generated higher fractions of both KLRG1+ CD8+ and CD4+ effector T-cells in the context of FVAX versus GVAX which may be significant in explaining the lack of cooperativity observed with GVAX (Figure S7). Also, it appeared that with GVAX the fraction of KLRG1+ CD4+ FoxP3- cells might be reduced in the combination treated mice relative to the mice receiving α4-1BB alone (mean of 31% vs. 23%); however, the magnitude of this difference did not reach statistical significance for the number of mice analyzed. We also found that although the numbers of KLRG1+ effectors per mm3 of tumor were higher with combination treatment versus α4-1BB alone, the numbers of highly-suppressive KLRG1+ Tregs were also increased (Figure S8). In addition to this increase of KLRG1+ Treg cells, we found far less advantageous effector T-cell to MDSC ratios in the tumors of mice treated with GVAX as opposed to FVAX (Figure 8). Also, the higher CD4+ to MDSC ratios observed with FVAX and αCTLA-4/α4-1BB versus α4-1BB alone were not evident in the context of GVAX. This increased suppressive burden in the context of GVAX versus FVAX could be reducing the fraction of tumor-infiltrating effector T-cells which develop into optimal effectors as well as reducing the cooperative augmentation of effector function we observe with FVAX.

Blockade of the co-inhibitory receptor CTLA-4 in the clinic has shown promise as a therapy for advanced solid tumors, however immune related adverse events associated with this treatment can be quite severe [24]. The major side of effect of 4-1BB agonist antibody treatment, at least in mice, appears to be an as yet poorly mechanistically defined inflammatory liver toxicity [10]. Interestingly, it has been reported in a pre-clinical model that αCTLA-4 and α4-1BB each reduced the auto-reactive side effects of the other [10]. Taken with our data showing strong cooperativity between these agents in rejecting B16-BL6 melanoma, further studies are certainly warranted to assess the safety of using 4-1BB agonist antibody and CTLA-4 blockade in combination.

## Materials and Methods

### Mice

All mouse procedures were performed in accordance with institutional protocol guidelines at Memorial Sloan-Kettering Cancer Center (MSKCC). Mice were maintained according to NIH Animal Care guidelines, under a protocol 04-07-019 approved by the MSKCC Institutional Animal Care Committee. C57BL/6 Mice (4–6 week old males) were obtained from Jackson Labs. B6.SJL mice (6 week old male) were obtained from Taconic.

### Antibodies

Anti-CTLA-4 (9D9), α4-1BB (LOB12.3) [12], Rat Ig and mIgG2b used *in vivo* were produced by Bioxcell. Dosing per injection was 100 ug 9D9, 350 ug LOB12.3, 350 ug RatIg, and 100 ug mIgG2b.

Staining antibodies included CD4-Q605, CD8-Pacific Orange, CD3-APCAlexa750 - Invitrogen. CD4-APC, FoxP3 Pacific Blue, KLRG1-APC, PD-1-FITC, 4-1BB-biotin - eBioscience. CD8-PE, IFN-γ-PE-CY7, Ki67-FITC, TNFα-APC - BD Bioscience. ICOS, CTLA-4 (4F10) - Bioxcell. CD45.1 PerCP - Biolegend. Some clones were conjugated using Invitrogen monoclonal antibody conjugation kits to either Alexa 532, Alexa 594, or Qdot 655.

### Cell Lines

B16/BL6 cells as well as B16-sFlt3L-Ig (FVAX) and B16-GMCSF (GVAX) have been described previously [4].

### Peptides

Ovalbumin 257–264 (SIINFEKL) and 323–339 (ISQAVHAAHAFINEAGR) (American Peptide Company) were used at a final concentration of 5 uM. GP100 (25), Trp-1 (455, 481, and 522), and Trp-2 (181) peptides used for re-stimulation of TIL were obtained from Biosynthesis Inc. and used at 10 uM final.

### B16 Melanoma Treatment Experiments

Mice were injected in the flank i.d. at day 0 with 2.5×10^4^ B16-BL6 cells and treated on days 3, 6, and 9 respectively with 1×10^6^ irradiated (150 Gy) gene-modified B16 cells on the contralateral flank and the indicated therapeutic antibody intra-peritoneally.

### Tumor Infiltration/Activation Marker Analysis

Mice receiving a 1.5×10^5^ B16-BL6 challenge were vaccinated as above on days 3,6, and 9 and sacrificied on Day 15. For Day-25 experiments, untreated and FVAX-treated mice received 2.5×10^4^ B16-BL6 cells, αCTLA-4 and α4-1BB treated mice received 1×10^5^ B16 cells, and combination treated mice received 2×10^5^ B16 cells with vaccination on days 4,7,10, and 13. Tumors were measured immediately prior to sacrifice. Excised tumors were digested in Liberase (Roche) and DNAase (Roche), cells were counted, and lymphocytes were enriched on a ficoll gradient (Sigma Histopaque 1119). Cells were stained using the eBioscience FoxP3 staining kit – CTLA-4 and PD-1 were stained for total intra- and extra-cellular protein. Stained samples were run completely on an LSRII (BD Bioscience) cytometer.

### Analysis of Cytokine Production

Mice receiving a 2.5×10^5^ B16-Ovalbumin challenge in 30% Matrigel (BD Bioscience) were vaccinated as above on days 6, 9 and 12 and sacrificied on Day 14. Vaccine and tumor draining lymph nodes as well as tumors were pooled from 5 mice per group. Lymph node cells were stained with DAPI and antibodies to CD4 and CD8 and sorted on a MoFlo cell sorter (Beckman Coulter). Tumor cells were purified as above except that prior to restimulation the T-cell fraction was enriched using a Miltenyi T-cell purification kit. 2×10^5^ CD8 cells and 2.5×10^5^ CD4 cells per well were restimulated in 96-well round bottom plates with 1×10^5^ Ovalbumin peptide pulsed DCs purified from B6.SJL spleens using CD11c positive selection beads (Miltenyi) for 36 hours and then cytokine production was assessed using the TH1/TH2/TH17 CBA kit from BD Bioscience. For tumor samples 2×10^6^ T-enriched TIL were re-stimulated with 7.5×10^5^ Ovalbumin and B16 melanoma peptide-pulsed DCs for 8 hours in the presence of GolgiPlug (BD Bioscience) and then fixed and stained for intra-cellular cytokine production using the eBioscience FoxP3 kit.

## Supporting Information

Figure S1 **Anti-CTLA-4 and α4-1BB antibodies alone do not cure B16-BL6.** Kaplan-Meier survival curves for mice challenged with 2.5×10^4^ B16-BL6 cells and vaccinated on days 3, 6 and 9 with the indicated antibody combination intra-peritoneally. Lack of survival was defined as death or tumor size >1000 mm^3^. Each curve represents 3 independent experiments of 5 mice per group. P values were calculated using the Log-rank (Mantel-Cox) test (* - p≤0.05, ** - p≤0.01, ***-p<0.001).
(TIF)

Figure S2 **Combination αCTLA-4/α4-1BB therapy drives high absolute numbers of CD4 and CD8 effectors to infiltrate tumors.** Mice were challenged with 1.5×10^5^ B16-BL6, treated with FVAX and the indicated antibody on days 3,6 and 9, and sacrificed on day 15. Number of lymphocytes per mm^3^ of tumor shown for A) CD8+ T-cells, B) CD4+ effector (Teff) and C) regulatory (Treg) cells. Values shown are for individually analyzed mice and are the sum of 5 independent experiments with 5–15 mice per group. Student's t-tests were performed to determine statistical significance between samples (* - p≤0.05, ** - p≤0.01, ***-p<0.001).
(TIF)

Figure S3 **Combination αCTLA-4/α4-1BB therapy promotes similar effector to regulatory T-cell ratios in the tumors of mice receiving GVAX compared to FVAX.** Mice challenged with 1.5×10^5^ B16-BL6 cells and treated on days 3, 6 and 9, were sacrificed on Day 15. TIL were fixed and stained for lymphocyte lineage and activation markers using the FoxP3 fixation kit. The ratios of A) CD8+ T-cells to Tregs and B) CD4+ Teff to Tregs are shown in the GVAX setting with means shown as green bars and with FVAX means shown in red for comparison. Values shown are for 10 individual mice per group.
(TIF)

Figure S4 **Combination αCTLA-4/α4-1BB increases T-cell IFN-γ production in tumor draining lymph node.** Mice challenged with 2.5×10^5^ B16-OVA cells and treated on days 6, 9 and 12, were sacrificed on Day 14. T-cells were purified from tumor-draining (TDLN), stained with antibodies, and sorted by flow cytometry into CD4+ and CD8+ subsets. Cytokine production was measured after 36 hours using the TH1/TH2/TH17 CBA Kit (BD) and is shown for 2×10^5^ TDLN CD8 T-cells restimulated on 1×10^5^ OVA 257–264 peptide pulsed DCs.
(TIF)

Figure S5 **Flow cytometry gating of tumor-infiltrating lymphocytes for cytokine production and proliferation.** Mice challenged with 2.5×10^5^ B16-OVA cells and treated on days 6,9 and 12, were sacrificed on Day 14. TIL were purified from 5–10 pooled tumors per group and enriched using the Miltenyi T-cell purification kit. 2×10^6^ TIL were restimulated with 7.5×10^5^ peptide-pulsed DC for 8 hours in the presence of BD GolgiPlug. Cells were fixed using the FoxP3 kit and analyzed by flow cytometry for lymphocyte markers, intracellular IFN-γ and TNF-α production, and Ki67 expression. Representative data is shown from one experiment to illustrate the gating and controls used to derive the data in Figure 4.
(TIF)

Figure S6 **4-1BB agonist antibody treatment reduces intra-tumoral FoxP3+ Treg proliferation.** Mice challenged with 2.5×10^5^ B16-OVA cells and treated on days 6, 9 and 12, were sacrificed on Day 14. TIL were purified from 5–10 pooled tumors per group and enriched using the Miltenyi T-cell purification kit. 2×10^6^ TIL were restimulated with 7.5×10^5^ peptide-pulsed DC for 8 hours in the presence of BD GolgiPlug. Cells were fixed using the FoxP3 kit and analyzed by flow cytometry for lymphocyte markers. Data shown is from 4 independent experiments for CD4+FoxP3+ Tregs. All means shown are +/− S.E.M.
(TIF)

Figure S7 **Combination αCTLA-4/α4-1BB therapy induces a higher fraction of CD8+ and CD4+ effector T-cells to become KLRG1+ with FVAX compared to GVAX.** Mice challenged with 1.5×10^5^ B16-BL6 cells and treated on days 3, 6 and 9, were sacrificed on Day 15. TIL were fixed and stained for lymphocyte lineage and activation markers using the FoxP3 fixation kit. The percentages of tumor-infiltrating A) CD8+ T-cells and B) CD4+ effector T-cells positive for KLRG1 are shown in the GVAX setting with means shown as green bars and with FVAX means shown in red for comparison. Values shown are for 10 individual mice per group.
(TIF)

Figure S8 **Combination αCTLA-4/α4-1BB therapy with GVAX induces higher tumor infiltration by both effector and regulatory KLRG1+ T-cells than α4-1BB alone.** Mice challenged with 1.5×10^5^ B16-BL6 cells and treated on days 3, 6 and 9, were sacrificed on Day 15. The number of KLRG1+ T-cells of a given lineage was calculated by multiplying the %KLRG1+ TIL determined by flow cytometry by the measured number of CD45+ lymphocytes per mm^3^ of tumor. Data shown are ratio of the absolute number of KLRG1+ T-cells from 10 combination treated tumors to the number of KLRG1+ T-cells from 10 α4-1BB alone treated tumors.
(TIF)

Figure S9 **Antibodies which block PD-1 or PD-L1 do not induce high levels of KLRG1 expression by tumor-infiltrating T-cells.** Mice challenged with 1.5×10^5^ B16-BL6 cells and treated on days 3, 6 and 9, were sacrificed on Day 15. TIL were fixed and stained for lymphocyte lineage and activation markers using the FoxP3 fixation kit. Percent of A) CD8+ T-cells, B) CD4+ effector (Teff) cells expressing KLRG1 are shown. Values shown are for individually analyzed mice and are the sum of 4–6 independent experiments with 5–15 mice per group. Student's t-tests were performed to determine statistical significance between samples(* - p≤0.05, ** - p≤0.01, ***-p<0.001).
(TIF)
