# Supplementary figures and images for: Combination CTLA-4 Blockade and 4-1BB Activation Enhances Tumor Rejection by Increasing T-Cell Infiltration, Proliferation, and Cytokine Production
Source: PLoS One. 2011 Apr 29;6(4):e19499. doi: 10.1371/journal.pone.0019499 (PMC3085474; doi:10.1371/journal.pone.0019499)

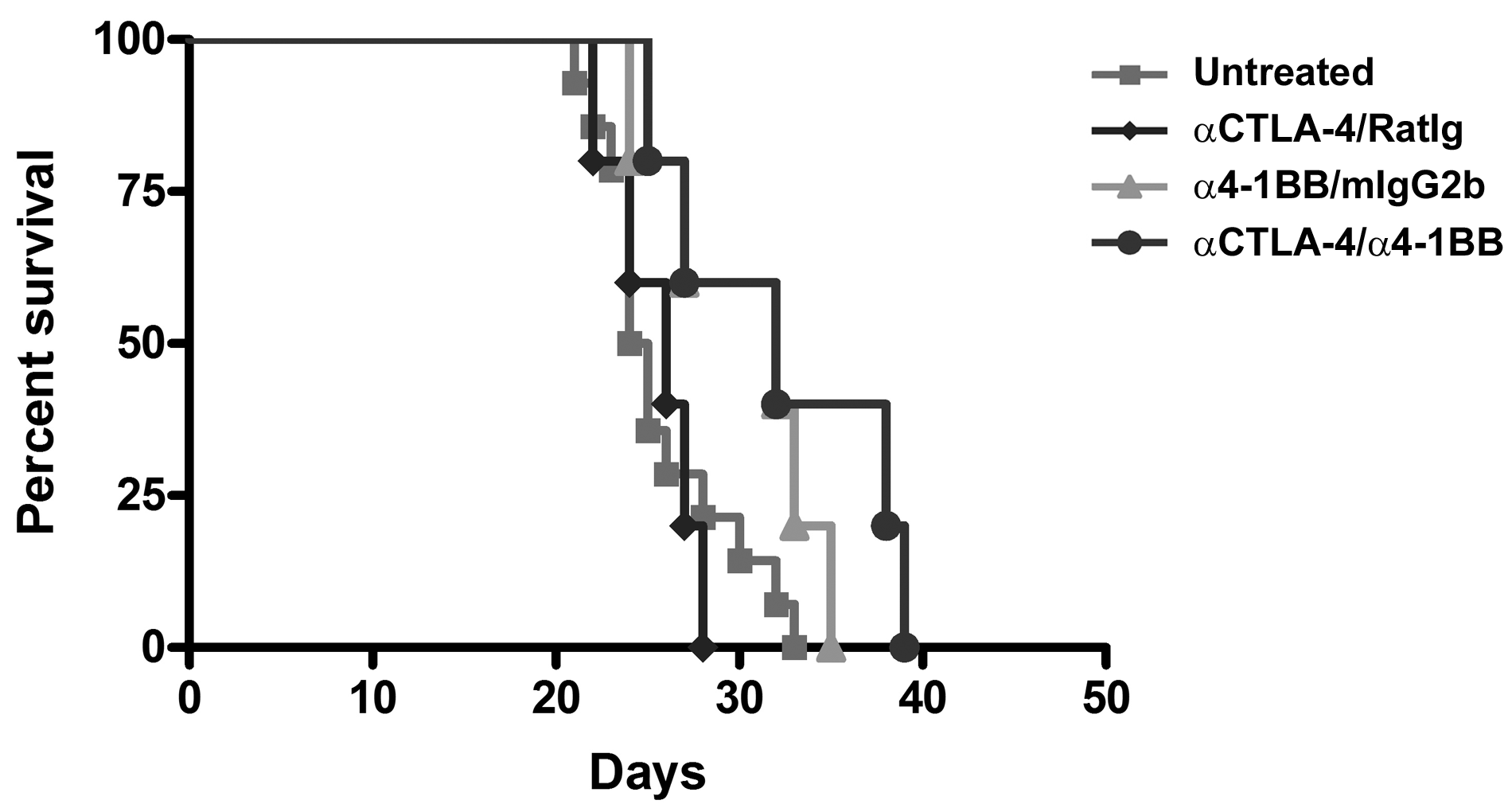

Supplement: Figure S1 — Anti-CTLA-4 and α4-1BB antibodies alone do not cure B16-BL6. Kaplan-Meier survival curves for mice challenged with 2.5×10^4^ B16-BL6 cells and vaccinated on days 3, 6 and 9 with the indicated antibody combination intra-peritoneally. Lack of survival was defined as death or tumor size >1000 mm3. Each curve represents 3 independent experiments of 5 mice per group. P values were calculated using the Log-rank (Mantel-Cox) test (* - p≤0.05, ** - p≤0.01, ***-p<0.001). (TIF) [file pone.0019499.s001.tif]

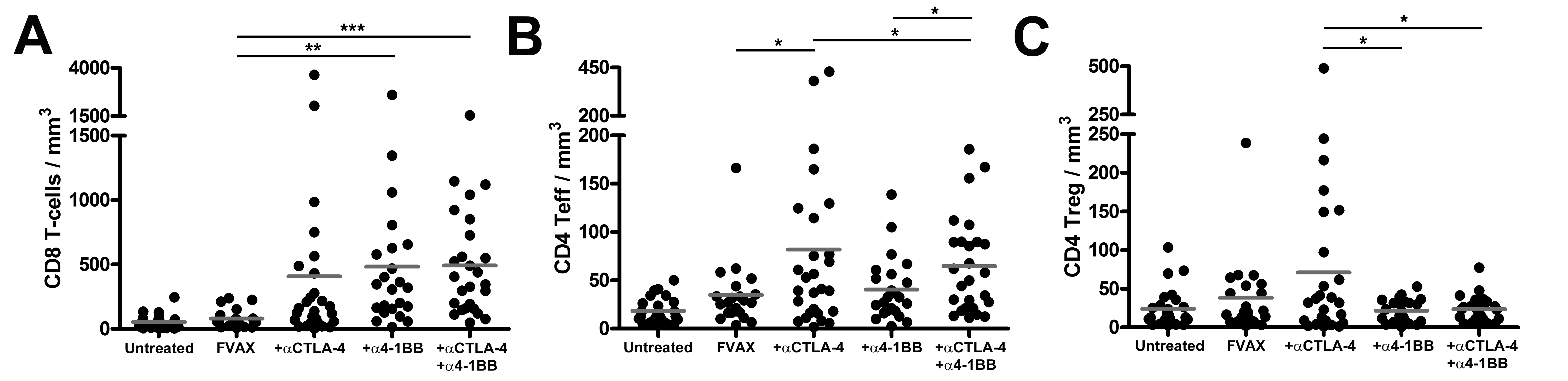

Supplement: Figure S2 — Combination αCTLA-4/α4-1BB therapy drives high absolute numbers of CD4 and CD8 effectors to infiltrate tumors. Mice were challenged with 1.5×10^5^ B16-BL6, treated with FVAX and the indicated antibody on days 3,6 and 9, and sacrificed on day 15. Number of lymphocytes per mm3 of tumor shown for A) CD8+ T-cells, B) CD4+ effector (Teff) and C) regulatory (Treg) cells. Values shown are for individually analyzed mice and are the sum of 5 independent experiments with 5–15 mice per group. Student's t-tests were performed to determine statistical significance between samples (* - p≤0.05, ** - p≤0.01, ***-p<0.001). (TIF) [file pone.0019499.s002.tif]

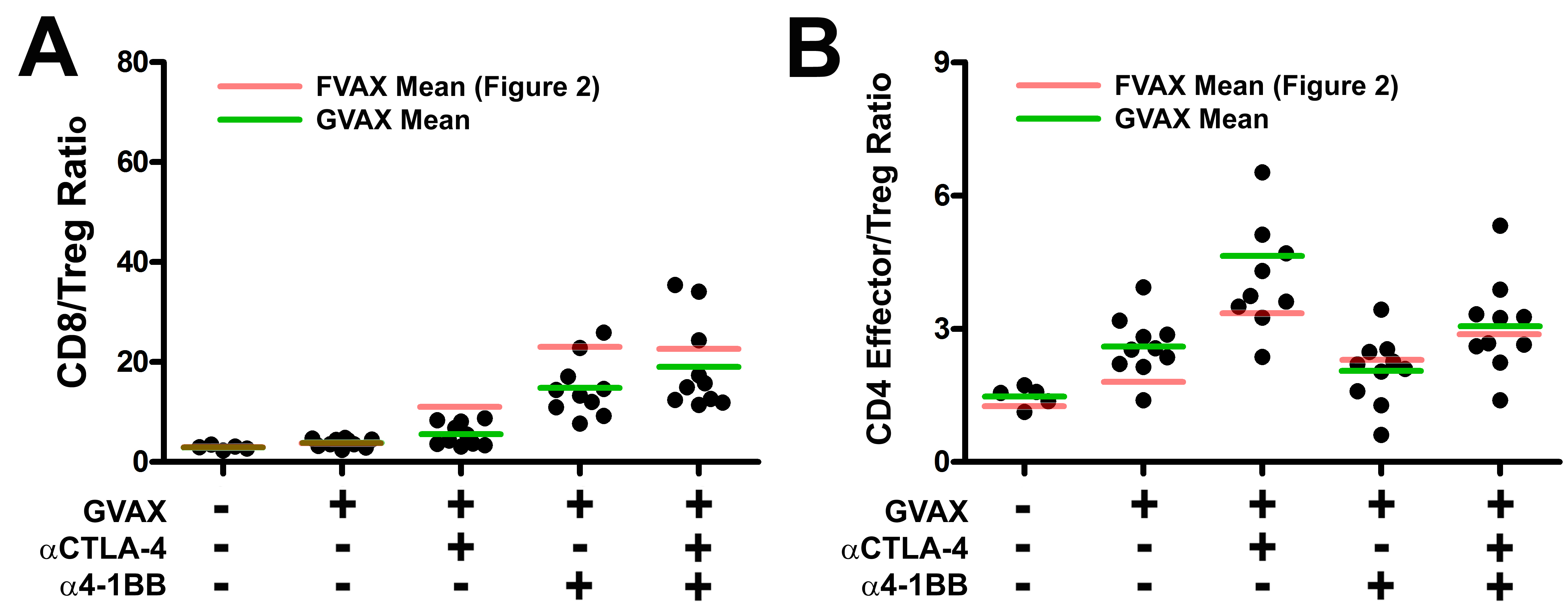

Supplement: Figure S3 — Combination αCTLA-4/α4-1BB therapy promotes similar effector to regulatory T-cell ratios in the tumors of mice receiving GVAX compared to FVAX. Mice challenged with 1.5×10^5^ B16-BL6 cells and treated on days 3, 6 and 9, were sacrificed on Day 15. TIL were fixed and stained for lymphocyte lineage and activation markers using the FoxP3 fixation kit. The ratios of A) CD8+ T-cells to Tregs and B) CD4+ Teff to Tregs are shown in the GVAX setting with means shown as green bars and with FVAX means shown in red for comparison. Values shown are for 10 individual mice per group. (TIF) [file pone.0019499.s003.tif]

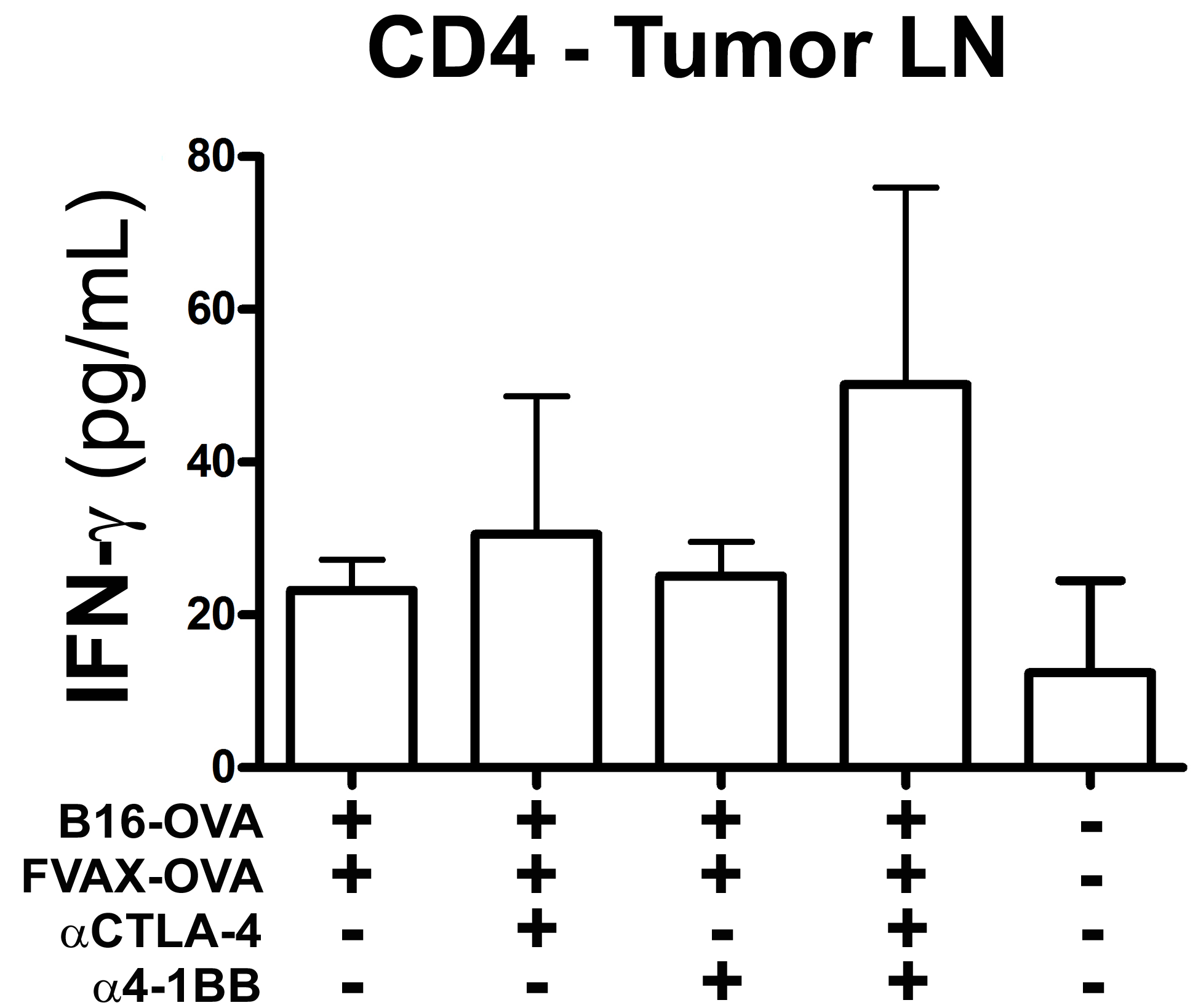

Supplement: Figure S4 — Combination αCTLA-4/α4-1BB increases T-cell IFN-γ production in tumor draining lymph node. Mice challenged with 2.5×10^5^ B16-OVA cells and treated on days 6, 9 and 12, were sacrificed on Day 14. T-cells were purified from tumor-draining (TDLN), stained with antibodies, and sorted by flow cytometry into CD4+ and CD8+ subsets. Cytokine production was measured after 36 hours using the TH1/TH2/TH17 CBA Kit (BD) and is shown for 2×10^5^ TDLN CD8 T-cells restimulated on 1×10^5^ OVA 257–264 peptide pulsed DCs. (TIF) [file pone.0019499.s004.tif]

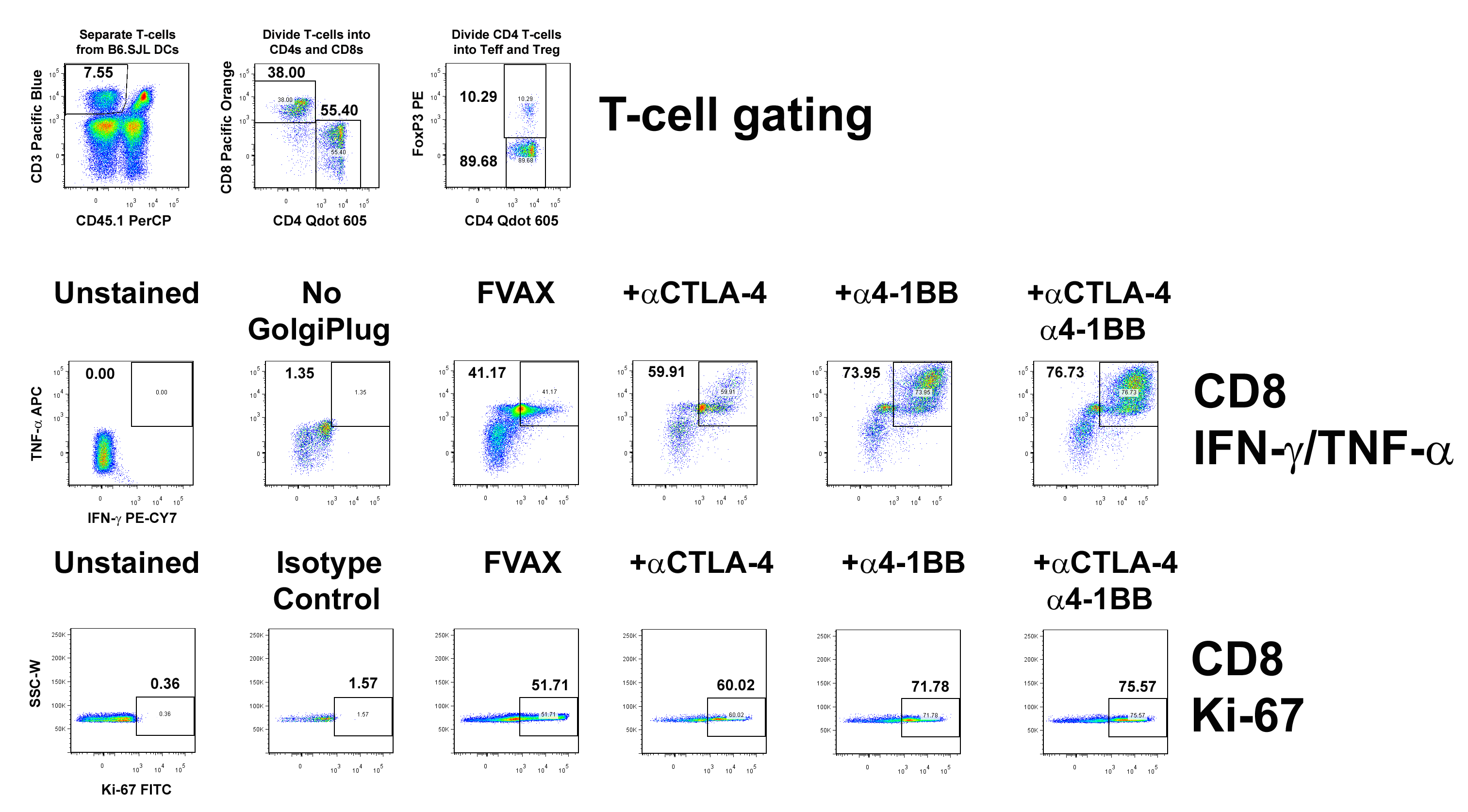

Supplement: Figure S5 — Flow cytometry gating of tumor-infiltrating lymphocytes for cytokine production and proliferation. Mice challenged with 2.5×10^5^ B16-OVA cells and treated on days 6,9 and 12, were sacrificed on Day 14. TIL were purified from 5–10 pooled tumors per group and enriched using the Miltenyi T-cell purification kit. 2×10^6^ TIL were restimulated with 7.5×10^5^ peptide-pulsed DC for 8 hours in the presence of BD GolgiPlug. Cells were fixed using the FoxP3 kit and analyzed by flow cytometry for lymphocyte markers, intracellular IFN-γ and TNF-α production, and Ki67 expression. Representative data is shown from one experiment to illustrate the gating and controls used to derive the data in Figure 4. (TIF) [file pone.0019499.s005.tif]

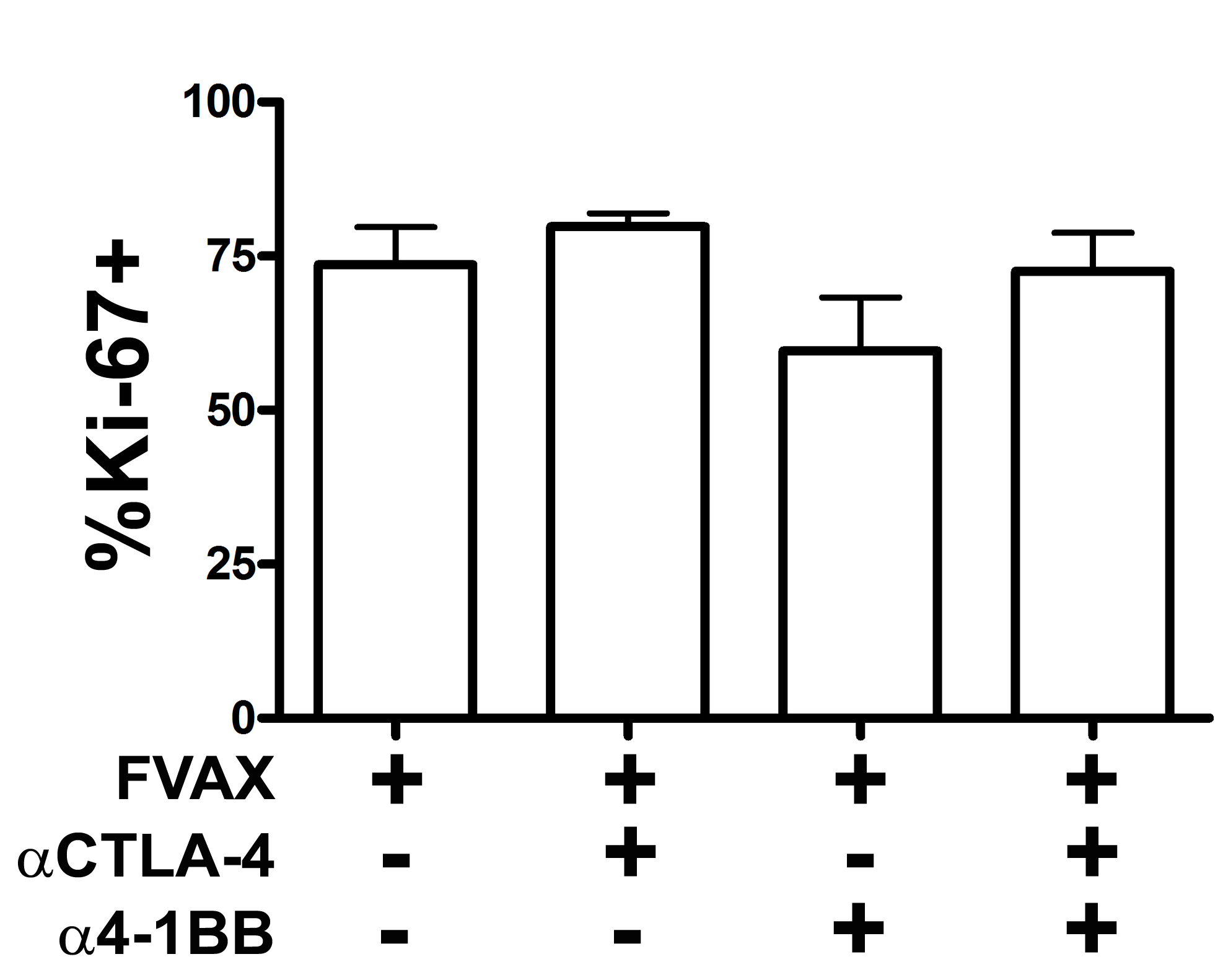

Supplement: Figure S6 — 4-1BB agonist antibody treatment reduces intra-tumoral FoxP3+ Treg proliferation. Mice challenged with 2.5×10^5^ B16-OVA cells and treated on days 6, 9 and 12, were sacrificed on Day 14. TIL were purified from 5–10 pooled tumors per group and enriched using the Miltenyi T-cell purification kit. 2×10^6^ TIL were restimulated with 7.5×10^5^ peptide-pulsed DC for 8 hours in the presence of BD GolgiPlug. Cells were fixed using the FoxP3 kit and analyzed by flow cytometry for lymphocyte markers. Data shown is from 4 independent experiments for CD4+FoxP3+ Tregs. All means shown are +/− S.E.M. (TIF) [file pone.0019499.s006.tif]

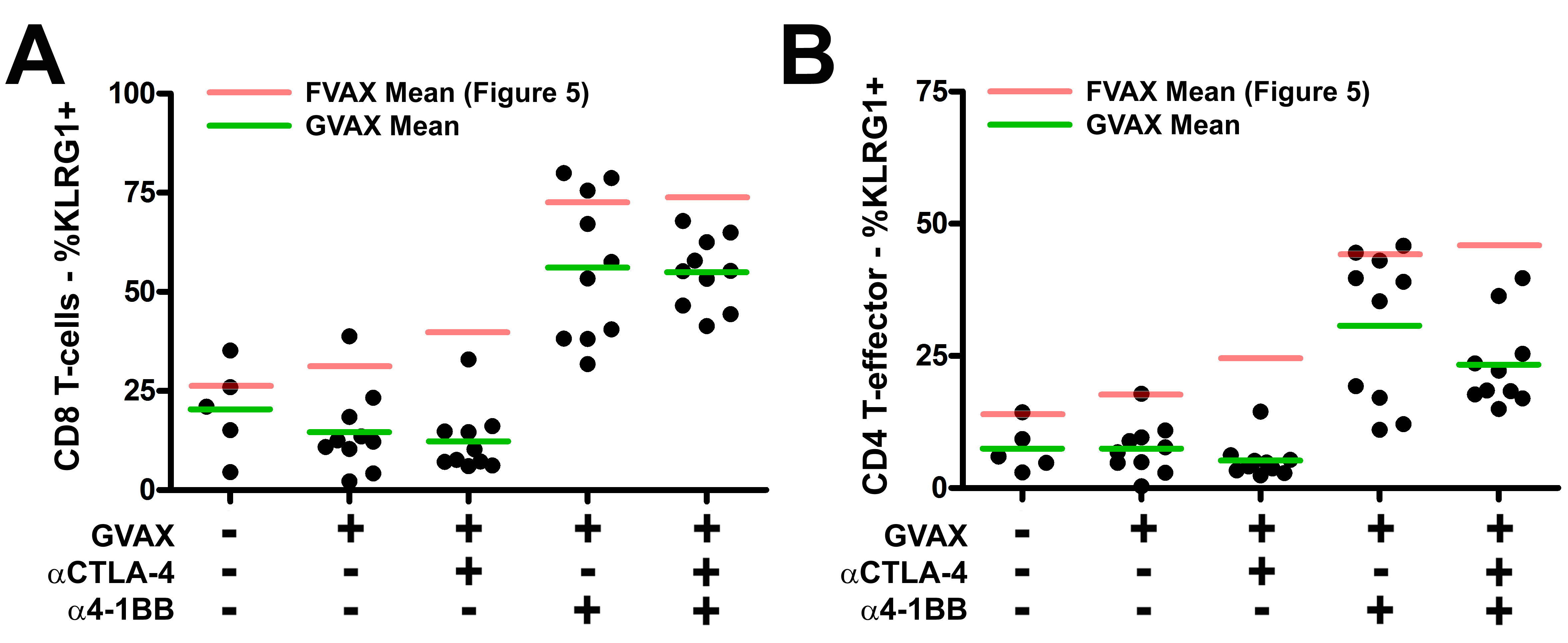

Supplement: Figure S7 — Combination αCTLA-4/α4-1BB therapy induces a higher fraction of CD8+ and CD4+ effector T-cells to become KLRG1+ with FVAX compared to GVAX. Mice challenged with 1.5×10^5^ B16-BL6 cells and treated on days 3, 6 and 9, were sacrificed on Day 15. TIL were fixed and stained for lymphocyte lineage and activation markers using the FoxP3 fixation kit. The percentages of tumor-infiltrating A) CD8+ T-cells and B) CD4+ effector T-cells positive for KLRG1 are shown in the GVAX setting with means shown as green bars and with FVAX means shown in red for comparison. Values shown are for 10 individual mice per group. (TIF) [file pone.0019499.s007.tif]

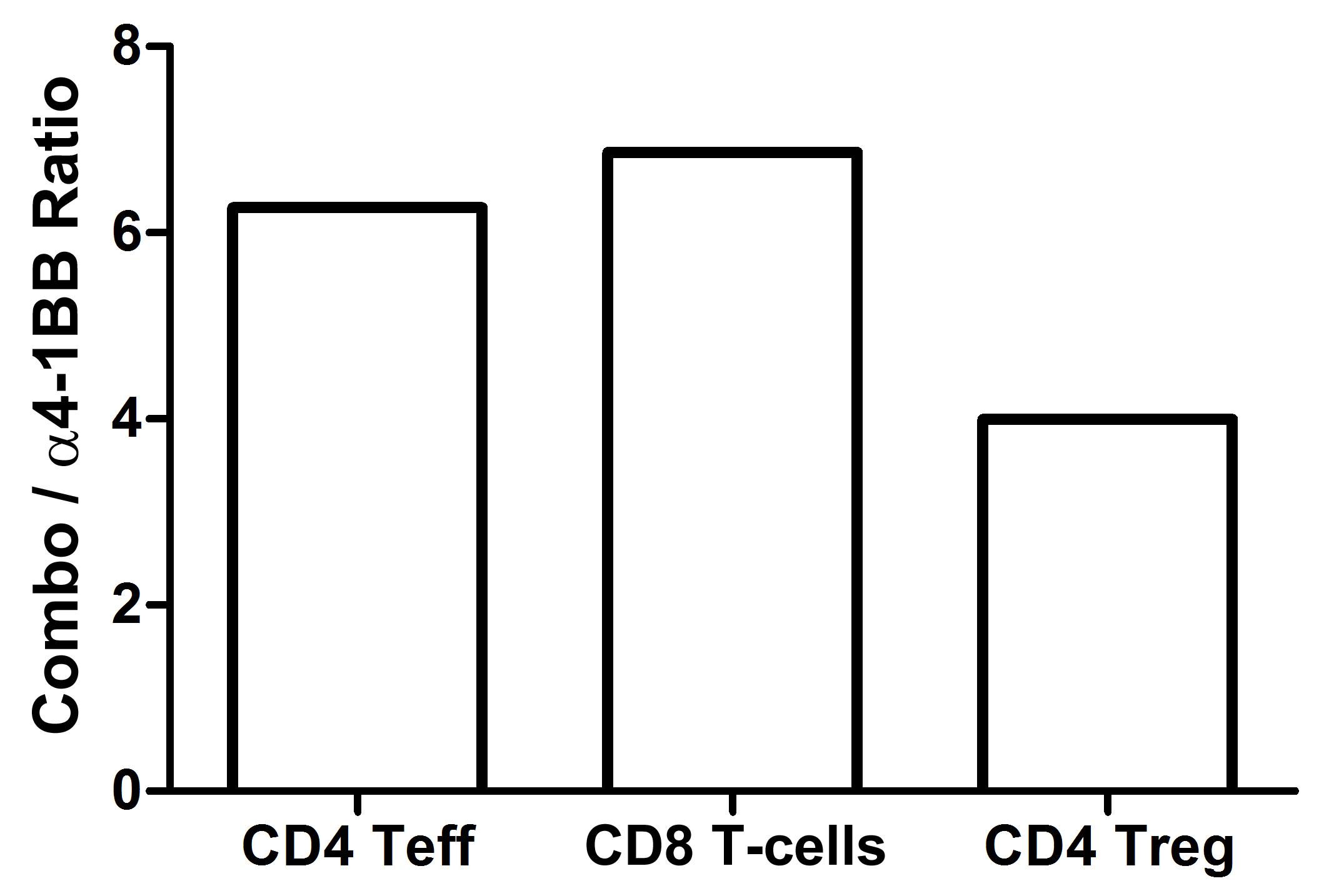

Supplement: Figure S8 — Combination αCTLA-4/α4-1BB therapy with GVAX induces higher tumor infiltration by both effector and regulatory KLRG1+ T-cells than α4-1BB alone. Mice challenged with 1.5×10^5^ B16-BL6 cells and treated on days 3, 6 and 9, were sacrificed on Day 15. The number of KLRG1+ T-cells of a given lineage was calculated by multiplying the %KLRG1+ TIL determined by flow cytometry by the measured number of CD45+ lymphocytes per mm3 of tumor. Data shown are ratio of the absolute number of KLRG1+ T-cells from 10 combination treated tumors to the number of KLRG1+ T-cells from 10 α4-1BB alone treated tumors. (TIF) [file pone.0019499.s008.tif]

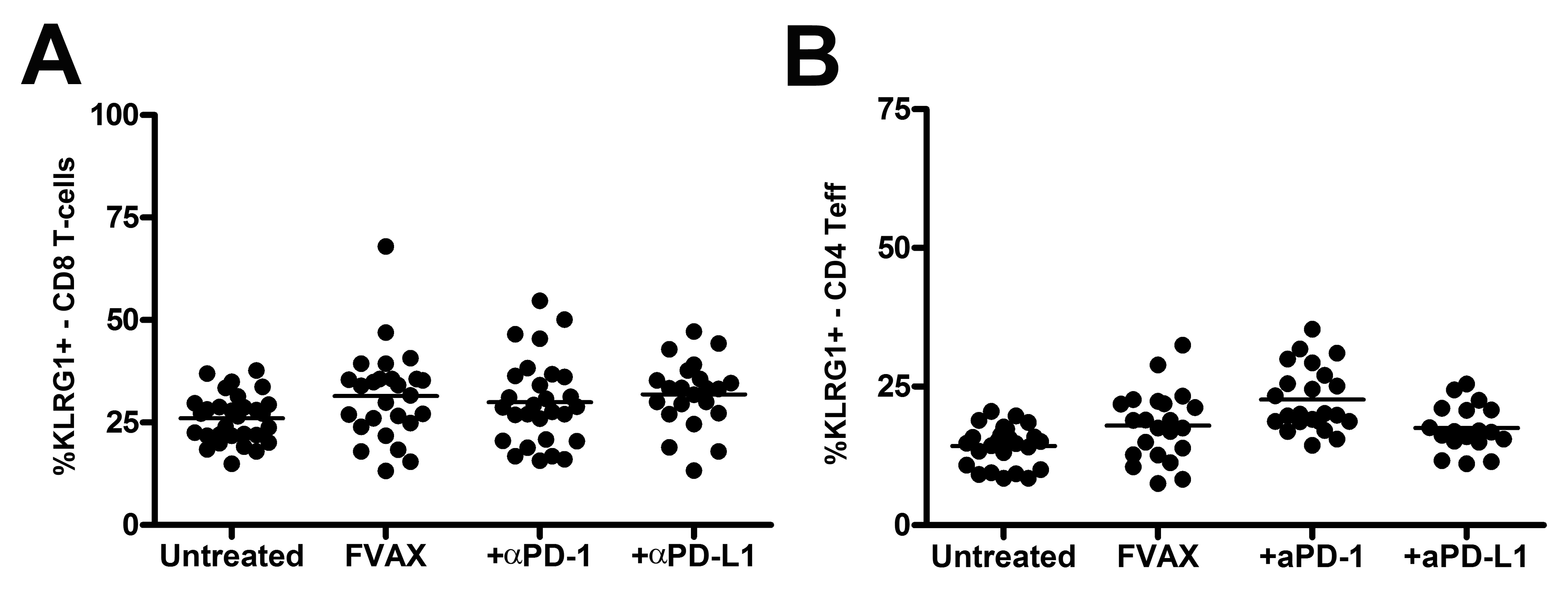

Supplement: Figure S9 — Antibodies which block PD-1 or PD-L1 do not induce high levels of KLRG1 expression by tumor-infiltrating T-cells. Mice challenged with 1.5×10^5^ B16-BL6 cells and treated on days 3, 6 and 9, were sacrificed on Day 15. TIL were fixed and stained for lymphocyte lineage and activation markers using the FoxP3 fixation kit. Percent of A) CD8+ T-cells, B) CD4+ effector (Teff) cells expressing KLRG1 are shown. Values shown are for individually analyzed mice and are the sum of 4–6 independent experiments with 5–15 mice per group. Student's t-tests were performed to determine statistical significance between samples(* - p≤0.05, ** - p≤0.01, ***-p<0.001). (TIF) [file pone.0019499.s009.tif]
